# Diagnostic performance of C-TIRADS in malignancy risk stratification of thyroid nodules: A systematic review and meta-analysis

**DOI:** 10.3389/fendo.2022.938961

**Published:** 2022-09-08

**Authors:** Yan Hu, Shangyan Xu, Weiwei Zhan

**Affiliations:** Department of Ultrasound, Ruijin Hospital, Shanghai Jiao Tong University School of Medicine, Shanghai, China

**Keywords:** meta-analysis, thyroid, thyroid nodules, ultrasound, risk assessment

## Abstract

**Background:**

Chinese thyroid imaging reports and data systems (C-TIRADS) is a novel malignancy risk stratification used for thyroid nodule diagnosis and guiding thyroid fine needle aspiration (FNA). In this review, we aim to assess the performance of C-TIRADS in malignancy risk stratification of thyroid nodules.

**Methods:**

PubMed, Medline, Web of Science, Embase, CNKI, and Wanfang databases were searched until 1 April 2022. Original articles reporting data about C-TIRADS and setting FNA or histology as reference standards were included. C-TIRADS 4A, 4B, and 4C were set as thresholds, respectively, to obtain pooled sensitivity, specificity, positive likelihood ratio (LR+), negative likelihood ratio (LR-), diagnostic odds ratio (DOR), and the area under the curve (AUC). Integrated nested Laplace approximation was used for Bayesian bivariate meta-analysis of diagnostic tests.

**Results:**

Sixteen studies were included, evaluating 11,506 thyroid nodules. The rate of malignancy in each risk classification is comparable with that in C-TIRADS. C-TIRADS 4B appeared to have better diagnostic performance than C-TIRADS 4A and 4C. The pooled sensitivity, specificity, LR+, LR-, and DOR of C-TI-RADS 4B were 0.94 (95% CI: 0.89–0.97), 0.70 (95% CI: 0.60–0.79), 3.20 (95% CI: 2.28–4.39), 0.09 (95% CI: 0.05–0.15), and 33.71 (95% CI: 25.51–42.40), respectively. The area under the summary ROC curve was 0.94 (95% CI: 0.90-0.96).

**Conclusion:**

C-TIRADS performed well in malignancy risk stratification of thyroid nodules. C-TIRADS 4B showed strong evidence of detecting malignancy.

## Introduction

Thyroid nodules are common. They are detected in 19%–67% of the population (1, 2). With a malignancy rate of less than 5%–10%, the purpose of evaluation for thyroid nodules is to identify malignant nodules (3–5). Ultrasound (US) has been widely applied in the initial evaluation of thyroid nodules and deemed an important standard to distinguish whether they are benign or malignant. A diagnosis based solely on US is not completely reliable (6), and the cytology obtained by fine needle aspiration (FNA) is still considered the gold standard diagnostic tool for thyroid nodules. Yet, the application of US-based risk stratification systems serves as a means to standardize the results of US examination and a tool for deciding which nodules should undergo FNA.

Previously, there have been several thyroid imaging reports and data systems (TIRADS), such as the American College of Radiology (ACR) TIRADS, the Korean (K) TIRADS, Kwak-TIRADS, and the European Thyroid Association (EU) TIRADS (7–10). In 2020, supported by the Superficial Organ and Vascular Ultrasound Group of the Society of Ultrasound in Medicine of the Chinese Medical Association and the Chinese Artificial Intelligence Alliance for Thyroid and Breast Ultrasound, Zhou et al. officially proposed a Chinese version of TIRADS (C-TIRADS) (11). C-TIRADS takes into account both the international standards for the US evaluation and the local conditions of the national health organization in China. Presently, C-TIRADS have been used in some studies to classify thyroid nodules (12–14), but the systematic performance of C-TIRADS has been so far marginally explored.

In this study, we aim to conduct a systematic review and meta-analysis to evaluate the performance of C-TIRADS.

## Materials and methods

### Search strategy and selection criteria

This meta-analysis was performed based on the Preferred Reporting Items for a Systematic Review and Meta-analysis (PRISMA) reporting guideline (15). We searched PubMed, Medline, Web of Science, Embase, CNKI, and Wanfang databases for studies published before 1 April 2022 using the following search terms: “Chinese-TIRADS,” “C-TIRADS,” “Chinese thyroid imaging reports and data systems,” and related terms.

The studies included in this analysis were based on the following criteria: (1) thyroid nodules were assessed by C-TIRADS classification; (2) reference standards were histopathological and/or cytological examination; (3) studies with sufficient data and without overlapping data were included; and (4) the search was limited to human studies published in English or Chinese. The full text was examined by two reviewers independently. Those that did not meet the criteria were excluded.

### Data collection and quality assessment

The following data were extracted from the main paper and supplementary data by two reviewers independently: (1) general information of the study (author, year of publication, study type, number of patients, sex distribution, average age/range of age, and number of nodules); (2) the reference standard for the diagnosis of malignancy; (3) the number of benign and malignant nodules; (4) the number of papillary thyroid carcinoma (PTC), follicular thyroid carcinoma (FTC), medullary thyroid carcinoma (MTC), and other malignancies; and (5) the US model and interpretation.

The risk of bias was assessed independently by two reviewers. The Quality Assessment of Diagnostic Accuracy Studies (QUADAS-2) tool was used for the following aspects: patient selection, index test, reference standard, flow, and timing (16). Risk of bias and concerns about applicability were assessed as low, high, or unclear. All the disagreements were resolved by two reviewers or adjudicated by a third reviewer.

### Assessment of thyroid nodules

Thyroid nodule assessment followed the C-TIRADS guideline (11), which excludes US features that have not been fully validated as risk factors for predicting malignancy. C-TIRADS assigned levels of malignancy risk to different patterns, a total of five features, namely solid composition, microcalcifications, markedly hypoechoic, ill-defined/irregular margins or extrathyroidal extensions, and vertical orientation. Each of these features scored +1 point. Comet-tail artifacts were considered as a sign of benign nodule and got -1 point. Every category and malignant rate were based on the points in C-TIRADS (**Table 1**).

**Table 1:** C-TIRADS malignancy risk stratification of thyroid nodules.

Category	US features	Points	Likelihood of malignancy
C-TIRADS 1	No nodule	-	0%
C-TIRADS 2	Benign	-1 point	0%
C-TIRADS 3	Probably benign	0 points	<2%
C-TIRADS 4A	Low suspicion for malignancy	1 point	2%–10%
C-TIRADS 4B	Moderate suspicion for malignancy	2 points	10%–50%
C-TIRADS 4C	High suspicion for malignancy	3–4 points	50%–90%
C-TIRADS 5	Highly suggestive of malignancy	5 points	>90%
C-TIRADS 6	Biopsy proved malignancy	-	100%

FNA was based on recommendations of C-TIRADS. The results of FNA were determined by the Bethesda system for reporting thyroid cytopathology (17). Class II was defined as benign and class V or VI as malignant. Class III and IV prompted a repeat FNA. When the repeat FNA was benign, the nodule was followed for 24 months or more, and if stable, it was classified as benign. Surgical histopathology, when available, was considered definitive.

### Evaluation of diagnostic accuracy

Meta-analysis was performed by R software (version 4.1.3) with the meta4diag and Bayesian bivariate integrated nested Laplace approximation (INLA) package (18). When we defined 4A as the cutoff, a benign nodule was considered as true negative if it was classified as C-TIRADS 2 or 3. A benign nodule was considered as false positive if it was classified as C-TIRADS 4A, 4B, 4C, or 5. A malignant nodule was considered as true positive if it was classified as C-TIRADS 4A, 4B, 4C, or 5. A malignant nodule was considered as false negative if it was classified as C-TIRADS class 2 or 3. With the same method, true negative, false positive, true positive, and false negative values were defined when setting 4B or 4C as the cutoff.

The diagnostic performance of C-TIRADS for thyroid nodules was analyzed with a random-effects model to calculate estimates of sensitivity, specificity, positive likelihood ratio (LR+), negative likelihood ratio (LR-), and diagnostic odds ratio (DOR) with 95% confidence intervals (95% CI), based on the extracted data of true positive, false positive, true negative, and false negative values. Forest plots of point estimates and 95% CI were provided. The DOR provides a single measure of test performance. Higher DOR values indicate better diagnostic performance. LR+ is the probability of biopsy-proven malignant nodules identified by high C-TIRADS classification (for example, when setting C-TIRADS 4A as the cutoff, C-TIRADS 4A, 4B, 4C, and 5 were regarded as high C-TIRADS classification) compared with that of benign nodules. LR+ higher than 10.0 means strong evidence; 5.0–10.0, moderate evidence; and less than 5.0, weak evidence. LR- is the probability of biopsy-proven benign nodules identified by low C-TIRADS classification (for example, when setting C-TIRADS 4A as the cutoff, C-TIRADS 2 and 3 were regarded as low C-TIRADS classification) compared with that of malignant nodules.

LR- less than 0.1 means strong evidence; 0.1–0.2, moderate evidence; and higher than 0.2, weak evidence. Crosshair plot and summary receiver-operating characteristic (SROC) curves were plotted by R software. Sensitivity analysis was used to evaluate the stability of the result of the meta-analysis *via* the sequential omission of individual studies.

## Results

### Search results

**Figure 1:**
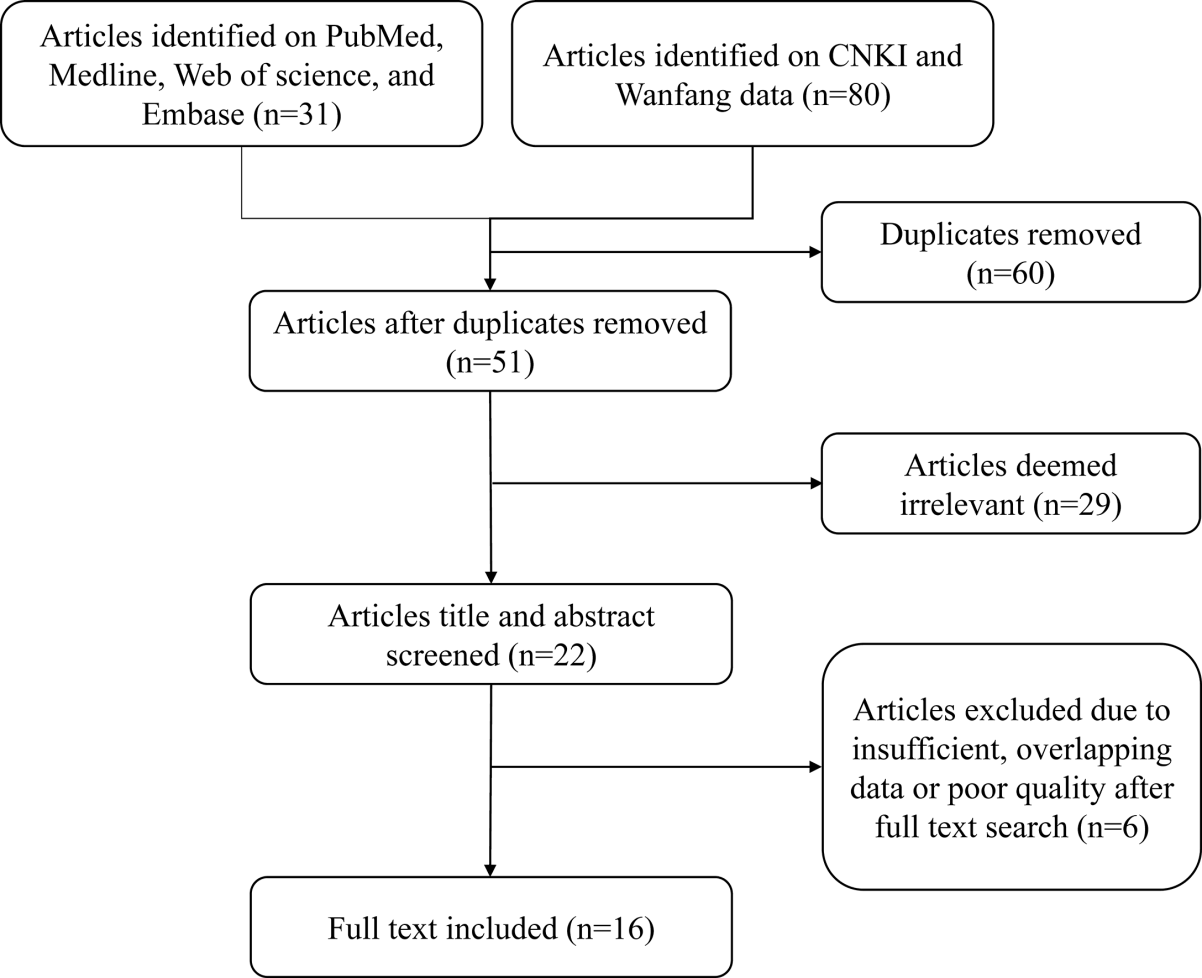
Flow diagram of the article selection process.

The initial search identified 111 articles from PubMed, Medline, Web of Science, Embase, CNKI, and Wanfang databases until 1 April 2022. After removing duplicates, we screened 51 articles through the title and the abstract, and 29 articles were deemed irrelevant. Following a full-text assessment, we removed 6 articles due to inadequate or overlapping data and poor quality. Eventually, 16 studies were selected for further analysis (**Figure 1**) (13, 14, 19–32). QUADAS-2 classification was used to assess the quality of included publications (**Figure S1**).

### Study and patient characteristics

There were 16 studies in total that included the data of 9,052 patients (**Table 1**). All the studies were retrospective in nature and were published between 2020 and 2022. The number of patients in each study varied between 70 and 2,141 (**Table 2**). In total, there were 6,820 women and 2,024 men. Of the 11,506 thyroid nodules included, 7,223 were benign and 4,283 were malignant (**Table 3**). The number of nodules varied from 92 to 2,141 in different studies. Histopathological and/or cytological evidence was regarded as the reference standard in all articles. If both histopathological and cytological examinations were available, the final diagnosis was based on histopathological results. According to nine studies that reported the type of malignant nodules, the most common subtype is papillary thyroid carcinoma.

**Table 2:** Baseline characteristics of included studies.

First author	Total no. of patients	Men	Women	Mean/median age	Interpretation	
					No. of readers	Average reader experience (y)
Cao 2021	355	99	256	49.7 ± 12.4	2	9
Fan 2021	759	144	615	49.0 ± 12.0	2	NA
Gao 2022	208	NA	NA	NA	2	NA
Li 2021	237	46	191	44.9 ± 11.5	2	NA
Li 2022	481	98	383	45.0 ± 10.4	2	8
Lin 2021	120	27	93	47.8 ± 12.4	1	5
Lin 2022	329	113	216	43.5 ± 14.3	2	NA
Qi 2021	884	203	681	49.26	4	5
Qiao 2021	433	82	351	46.6 ± 12.9	2	NA
Sui 2021	70	13	57	48.1 ± 11.5	2	NA
Wu 2021	104	30	74	NA	2	10+
Zhang 2021	408	93	315	NA	NA	NA
Zhang 2022	560	132	428	47.5 ± 13.0	3	5
Zheng 2021	266	70	196	43.9 ± 12.6	4	10
Zhou 2020	2,141	513	1628	50.3 ± 12.0	4	10
Zhu 2021	1,697	361	1336	49.7 ± 12.2	2	15
**Total**	9,052	2,024	6820	–	–	–

NA, not applicable.

**Table 3:** Characteristics of thyroid nodules included.

First author	Total no. of nodules	Total no. of malignant nodules	Total no. of benign nodules	Median/mean nodule size (range) (cm)	Reference standard		Type of malignant nodule				
					Surgery	Biopsy	PTC	FTC	MTC	Undifferentiated carcinoma	Other
Cao 2021	388	233 (60.1%)	155 (39.9%)	1.39 ± 0.85 (0.4–4.8)	Yes	NA	229 (98.3%)	1 (1.3%)	3 (1.3%)	0	0
Fan 2021	2213	490 (22.1%)	1723 (77.9%)	1.1 ± 0.8	Yes	NA	NA	NA	NA	NA	NA
Gao 2022	251	132 (52.6%)	119 (47.4%)	NA	Yes	NA	126 (95.5%)	0	4 (3.0%)	1 (0.8%)	1 (0.8%)
Li 2021	237	132 (55.7%)	105 (44.3%)	1.42 ± 0.63 (0.3–3.0)	Yes	Yes	NA	NA	NA	NA	NA
Li 2022	513	206 (40.2%)	307 (59.8%)	2.74 ± 1.14	Yes	NA	187 (90.8%)	13 (6.3%)	2 (1.0%)	2 (1.0%)	2 (1.0%)
Lin 2021	123	67 (54.5%)	56 (45.5%)	1.29 ± 1.15 (0.2–7.5)	Yes	Yes	65 (97.0%)	1 (1.5%)	0	0	1 (1.5%)
Lin 2022	329	67 (20.4%)	262 (79.6%)	3.6 ± 1.7	Yes	NA	0	67 (100%)	0	0	0
Qi 2021	1096	414 (37.8%)	682 (62.2%)	1.9 (0.5–6.4)	Yes	Yes	384 (92.8%)	10 (2.4%)	7 (1.7%)	6 (1.5%)	7 (1.7%)
Qiao 2021	433	202 (46.7%)	231 (53.3%)	1.13 ± 0.55	Yes	Yes	NA	NA	NA	NA	NA
Sui 2021	92	50 (54.3%)	42 (45.7%)	1.42 ± 0.98 (0.32–4.1)	Yes	NA	47 (94.0%)	1 (2.0%)	2 (4.0%)	0	0
Wu 2021	104	66 (63.5%)	38 (36.5%)	NA	NA	Yes	NA	NA	NA	NA	NA
Zhang 2021	434	187 (43.1%)	247 (56.9%)	NA	Yes	Yes	NA	NA	NA	NA	NA
Zhang 2022	560	370 (66.1%)	190 (33.9%)	0.5-5.4	Yes	Yes	NA	NA	NA	NA	NA
Zheng 2021	283	211 (74.6%)	72 (25.4%)	NA	Yes	Yes	NA	NA	NA	NA	NA
Zhou 2020	2,141	565 (26.4%)	1,576 (73.6%)	2.33 ± 1.43 (0.23–8.60)	Yes	NA	529 (93.8%)	14 (2.5%)	21 (3.7%)	0	0
Zhu 2021	2,309	891 (38.6%)	1,418 (61.4%)	1.31 ± 1.06 (0.02–6.9)	Yes	Yes	800 (99.1%)	4 (0.5%)	2 (0.2%)	1 (0.1%)	0
**Total**	**11,506**	**7,223**	**4,283**	–	–	–	–	–	–	–	–

PTC, papillary thyroid carcinoma; FTC, follicular thyroid carcinoma; MTC, medullary thyroid carcinoma.

NA, not applicable.

### Diagnostic performance of C-TIRADS in thyroid nodule assessment

Firstly, we calculated the prevalence of malignancy in each risk stratification category. The rate of malignant thyroid nodules was 0% in C-TIRADS 2, 1.37% in C-TIRADS 3, 10.62% in C-TIRADS 4A, 40.02% in C-TIRADS 4B, 77.96% in C-TIRADS 4C, and 94.61% in C-TIRADS 5 (**Table 4**).

**Table 4:** The prevalence of malignancy in each C-TIRADS classification.

Classification	No. of malignant nodules	Total no. of nodules	Prevalence of malignancy (%)	Suggested malignancy risk (%)
C-TIRADS 2	0	370	0%	0%
C-TIRADS 3	31	2,271	1.37%	<2%
C-TIRADS 4A	301	2,834	10.62%	2–10%
C-TIRADS 4B	854	2,134	40.02%	10–50%
C-TIRADS 4C	2,763	3,544	77.96%	50–90%
C-TIRADS 5	334	353	94.62%	>90%

C-TIRADS, Chinese thyroid imaging reports and data systems.

**Figure 2:**
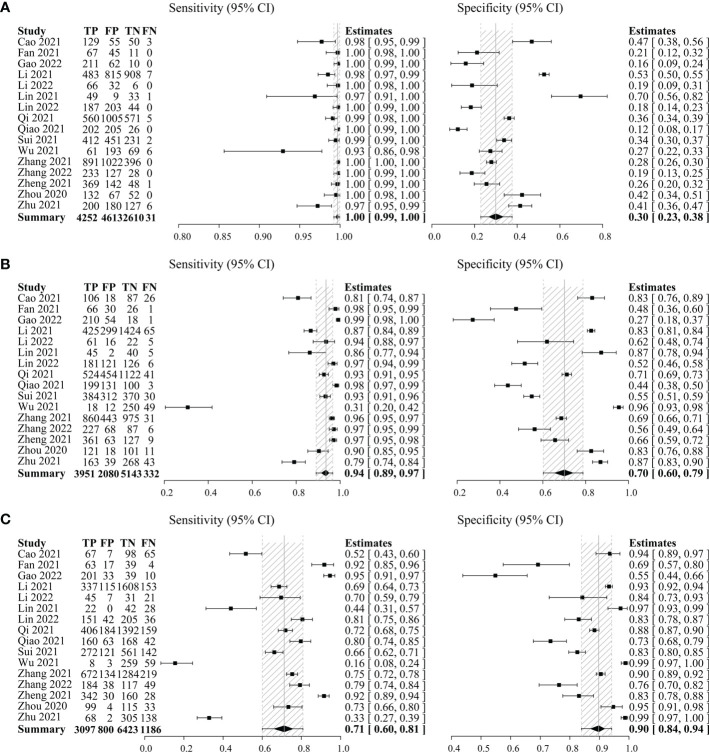
Forest plots with individual and pooled sensitivity and specificity of C-TIRADS 4A **(A)**, 4B **(B)**, and 4C **(C)** in the evaluation of thyroid nodules. The estimated accuracy for each study is plotted as a point and the 95% confidence interval (CI) as arrows. TP, true positivity; FP, false positivity; TN, true negativity; FN, false negativity; C-TIRADS, Chinese thyroid imaging reporting and data system.

Secondly, C-TIRADS 4A, 4B, and 4C were each analyzed separately to get the diagnostic indicators. The pooled sensitivity of C-TIRADS 4A (1.00, 95% CI: 0.99–1.00) was higher than 4B (0.94, 95% CI: 0.89–0.97) and 4C (0.71, 95% CI: 0.60–0.81), while the pooled specificity of C-TIRADS 4C (0.90, 95% CI: 0.84–0.94) was higher than 4A (0.30, 95% CI: 0.23–0.38) and 4B (0.70, 95% CI: 0.60–0.79) (**Figure 2**).

**Figure 3:**
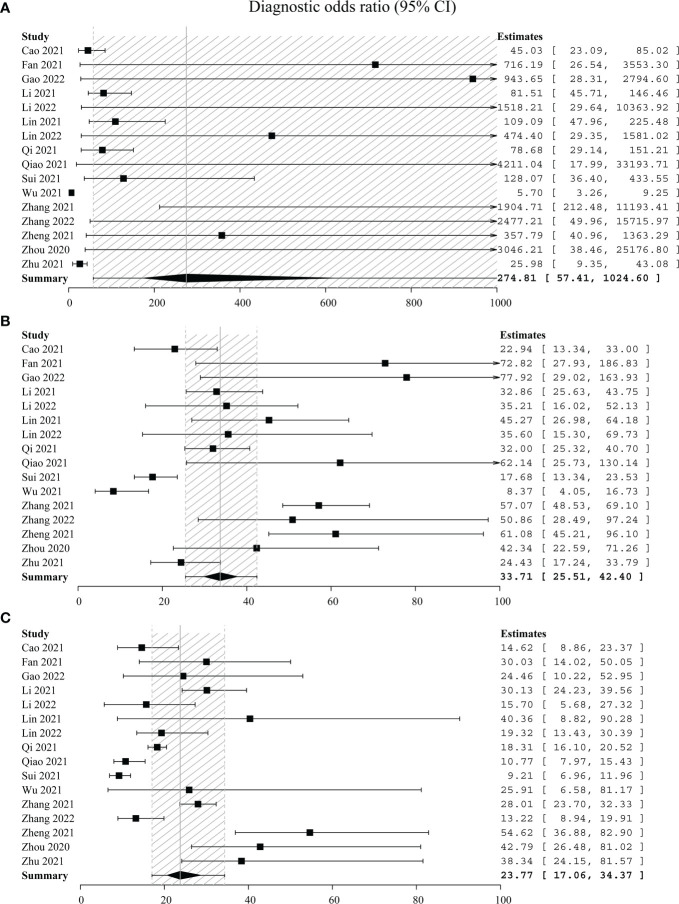
Forest plots with individual and pooled DOR of C-TIRADS 4A **(A)**, 4B **(B)**, and 4C **(C)**. The estimated accuracy for each study is plotted as a point and the 95% confidence interval (CI) as arrows. DOR, diagnostic odds ratio; C-TIRADS, Chinese thyroid imaging reporting and data system.

**Figure 4:**
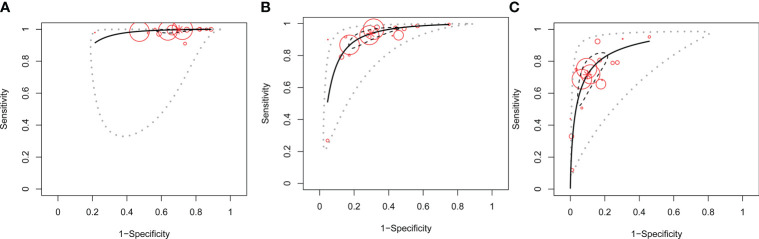
SROC of C-TIRADS 4 in detecting malignancy. SROC analysis showing the diagnostic performance of C-TIRADS 4A **(A)**, 4B **(B)**, and 4C **(C)**. The summary point is indicated by the red star; each individual study is represented by red circles (scale = study sample size). The area enclosed by the inner (black line) and outer (gray line) ellipses represents the confidence region and the prediction region of the summary points. SROC, summary receiver operating curve; C-TIRADS, Chinese thyroid imaging reporting and data system.

Thirdly, DOR and the SROC plot were used to determine the optimal one between C-TIRADS 4B and 4C. The DOR of C-TIRADS 4B ranged from 8.37 to 77.92 (summary 33.71, 95% CI: 25.51–42.40), while C-TIRADS 4C ranged from 9.21 to 54.62 (summary 23.77, 95% CI: 17.06–34.37) (**Figure 3**). The SROC plots suggested that the AUC of 4B (0.94, 95% CI: 0.90–0.96) was higher than that of 4C (0.89, 95% CI: 0.84–0.92) (**Figure 4**; **Table 5**). These results indicated that C-TIRADS 4B was superior to 4C in detecting malignancy.

**Table 5:** Summary estimates of the diagnostic performance of C-TIRADS.

Classification	LR+ (95% CI)	LR- (95% CI)	AUC (95% CI)
C-TIRADS 4A	1.43 (1.28–1.63)	0.01 (0.00–0.03)	1.00 (0.99–1.00)
C-TIRADS 4B	3.20 (2.28–4.39)	0.09 (0.05–0.15)	0.94 (0.90–0.96)
C-TIRADS 4C	7.38 (4.54–12.00)	0.32 (0.20–0.45)	0.88 (0.82–0.92)

C-TIRADS, Chinese thyroid imaging reports and data systems; LR+, positive likelihood ratio; LR-, negative likelihood ratio; AUC, area under curve; 95% CI, 95% confidence interval.

To further evaluate the diagnostic performance of C-TIRADS 4B, LR+ was 3.20 (95% CI: 2.28–4.39) and LR- was 0.09 (95% CI: 0.05–0.15) (**Table 5**). These provided strong evidence for 4B to differentiate malignant nodules.

### Evaluation of study heterogeneity

**Figure 5:**
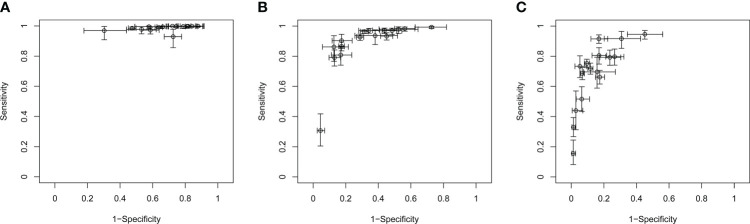
Crosshair plots of the sensitivity and specificity. Crosshair plots showing individual sensitivity and specificity of C-TIRADS 4A **(A)**, 4B **(B)**, and 4C **(C)** in the assessment of thyroid nodules. The estimated accuracy for each study is plotted as a circle, and 95% CI is plotted as arrows. C-TIRADS, Chinese thyroid imaging reporting and data system.

Study heterogeneity was assessed with crosshair plots and sensitivity analysis. Crosshair plots were made to show the scatter of the study results (**Figure 5**). There were no significant differences among the sensitivity of 16 included studies, while the specificities were quite different from each other with a wide interval. To investigate the influence of a single study on the overall analysis, we omitted one study at a time. The omission of any study did not significantly change the corresponding pooled sensitivity, specificity, LR+, LR-, DOR, and AUC (**Table S1**). Both sensitivity analysis and crosshair plots indicated that our results were robust and reliable.

## Discussion

This systematic review is, to our knowledge, the first to consider all available data using a meta-analytic approach, confirmed by a search of database, thus representing the first review of C-TIRADS internationally. We collected and analyzed 16 articles involving a total of 11,506 nodules (7,223 benign, 4,283 malignant) to assess the diagnostic performance of C-TIRADS in malignancy risk stratification of thyroid nodules. We investigated whether the malignancy rate observed in this analysis was consistent with that of the C-TIRADS guideline. Moreover, a series of diagnostic indicators were used to evaluate the performance of C-TIRADS by setting 4B as the cutoff. We believe that this analysis can provide more convincing evidence and support for wider application and deeper understanding of C-TIRADS.

The rate of malignant thyroid nodules was 0% (0%) in C-TIRADS 2, 1.37% (< 2%) in C-TIRADS 3, 10.62% (2–10%) in C-TIRADS 4A, 40.02% (10–50%) in C-TIRADS 4B, 77.96% (50–90%) in C-TIRADS 4C, and 94.61% (> 90%) in C-TIRADS 5 (**Table 4**). These results compared favorably with the C-TIRADS guideline designation of “likelihood of malignancy” (11). C-TIRADS should be generally considered as an accurate system to stratify the risk of malignancy of thyroid nodules.

Our results show the high accuracy of the C-TIRADS 4B class in the detection of thyroid malignancies. In facts, C-TIRADS 4B detected 94% of malignant nodules while misdiagnosed 30% of benign nodules as suspicious. Similar with 4C and 5 nodules, C-TIRADS 4B nodules do require FNA as recommended by C-TIRADS guideline (11). In those 4B nodules presenting with a negative FNA result, a second FNA could be performed to confirm their benign nature. The data obtained in our study may raise the question whether a binary high- *vs*. low-risk stratification of thyroid nodules could be regarded as sufficiently accurate for selecting patients to be referred to FNA and possibly to surgical treatment. With an acceptably low rate (< 2%) of false negative results, C-TIRADS 2 and 3 classes could perhaps both be included in a benign/likely benign single class. Instead, the malignant risk of C-TIRADS 4A (10.62%) is too high to consider the inclusion of nodules belonging to this category in the “benign” subgroup. At the same time, the C-TIRADS 4A class does not qualify a nodule as likely malignant since the risk of malignancy in this category (5–10%) is similar to the one recorded in the general population (3–5). As expected, C-TIRADS 4A included a majority of benign nodules. Hence, given the high proportion of nodules included in this class (2,834/11,506), a substantial burden for the patients and the health care system could be generated if all TIRADS 4A nodules are referred to FNA (and possibly to subsequent surgery). Yet, the frequency of malignant nodules in this class is too high to be neglected. Hence, the management of thyroid nodules classified as 4A should take into account other clinical risk factors such as large size, isthmic or upper lobe location, and positive, family history (33–35), as also recommended in the C-TIRADS guidelines (11). In addition, since the potential of malignancy is higher in iodine-sufficient areas, the management of C-TIRADS 4A nodules could be determined from region to region based on local iodine sufficiency (or deficiency).

It is obvious that the binary organization of C-TIRADS may not be sufficient to exclude a suspicion of malignancy if a thyroid nodule is diagnosed as C-TIRADS 4A, despite the fact that using C-TIRADS 4B as a cutoff showed excellent diagnostic performance for malignant nodules. Tertiary organization of C-TIRADS, for which C-TIRADS 4A can be considered the intermediate-risk class, may be useful in the management of thyroid nodules. Thyroid nodules classified as intermediate-risk class should be treated more effectively in conjunction with clinical factors, such as more frequent ultrasound surveillance than low-risk stratification (C-TIRADS 2 and 3) and delayed FNA testing than high-risk stratification (C-TIRADS 4B, 4C, and 5). After risk assessment, FNA is the next step in the triage of a thyroid nodule. It should be reserved for lesions that have been determined to be sufficiently suspicious based on C-TIRADS risk stratification. The outcomes are critical in optimizing subsequent management. FNA molecular testing is a new approach that may reduce the need for diagnostic surgery (35). Targeted next-generation sequencing analysis of cancer-related genes for point mutations, gene fusions, copy number alterations, or abnormal gene expression is among the tests developed for this purpose (36). However, molecular testing should unquestionably be taken into consideration if clinical, imaging, and cytology results are insufficient for diagnosis and surgery is the only diagnostic option (5, 37).

This analysis indicates that C-TIRADS performs well in malignancy risk stratification of thyroid nodules and provides more support for appropriate use of FNA recommended by C-TIRADS. Moreover, periodic revisions and updates of C-TIRADS, mainly based on solid evidence and new studies, are necessary to comprehensively reflect the risks and guide FNA. There is no large prospective study evaluating C-TIRADS so far. Further studies are needed to better guide clinical practice.

The diagnostic performance of C-TIRADS was compared with other risk stratification systems in the following 4 publications. Zhou et al. (32) evaluated 2,141 nodules and demonstrated that the diagnostic efficacy of C-TIRADS was significantly greater than that of the American Thyroid Association (ATA) guidelines, the American College of Radiology (ACR) TIRADS, and the Korean TIRADS. Zhu et al. (25) also found that C-TIRADS had better diagnostic performance and a relatively lower unnecessary biopsy rate in detecting thyroid cancer compared to the other three guidelines. On the other hand, the results of Qi et al. (13), which analyzed 3,524 nodules, showed that C-TIRADS had only a little advantage over the ACR TIRADS and the K-TIRADS, and a significant advantage over the EU-TIRADS. This may be due to sample size limitations and bias caused by the fact that not all patients meeting the criteria were included in the study. Furthermore, Zhou et al. (32) found that the EU-TIRADS and ATA guidelines did not apply to 5.1% and 9.9% of nodules, whereas C-TIRADS applied to all nodules.

There are also several limitations that need to be considered. Firstly, all the studies included were retrospective in nature. There was concern for poor US image quality, and retrospective review may have led to wrong classification. Secondly, the nodule size is another important factor for FNA. However, only two articles reported the nodule size in each classification. Thus, the deviation may affect the risk of FNA in the current study. Thirdly, PTC accounts for more than 90% of current reports. Further research is needed to evaluate the diagnostic performance of C-TIRADS in specific subtypes of thyroid cancer.

## Conclusion

In conclusion, C-TIRADS is a good tool for malignancy risk stratification of thyroid nodules. This review provides strong evidence for C-TIRADS 4B in the assessment of malignant thyroid nodules. Further validation of this tool is required.

## Data availability statement

The original contributions presented in the study are included in the article/supplementary material. Further inquiries can be directed to the corresponding author.

## Author contributions

WZ conceived the meta-analysis. All authors contributed to the development of the selection criteria, the risk of bias assessment strategy, and data extraction criteria. YH developed the search strategy. YH and SX performed the database search, acquired the data, and analyzed the data. YH and WZ drafted the manuscript. All authors contributed to the article and approved the submitted version.

## Funding

The study was funded by the National Natural Science Foundation of China (82071923).

## Acknowledgments

The authors would like to thank their colleagues and institutions for their great assistance in this study.

## Conflict of interest

The authors declare that the research was conducted in the absence of any commercial or financial relationships that could be construed as a potential conflict of interest.

## Publisher’s note

All claims expressed in this article are solely those of the authors and do not necessarily represent those of their affiliated organizations, or those of the publisher, the editors and the reviewers. Any product that may be evaluated in this article, or claim that may be made by its manufacturer, is not guaranteed or endorsed by the publisher.
