# Supplementary material for: Diagnostic performance of C-TIRADS in malignancy risk stratification of thyroid nodules: A systematic review and meta-analysis
Source: Front Endocrinol (Lausanne). 2022 Sep 8;13:938961. doi: 10.3389/fendo.2022.938961 (PMC9492922; doi:10.3389/fendo.2022.938961)
Supplement: Supplementary file 1 [file Image_1.pdf]

Supplementary Figures

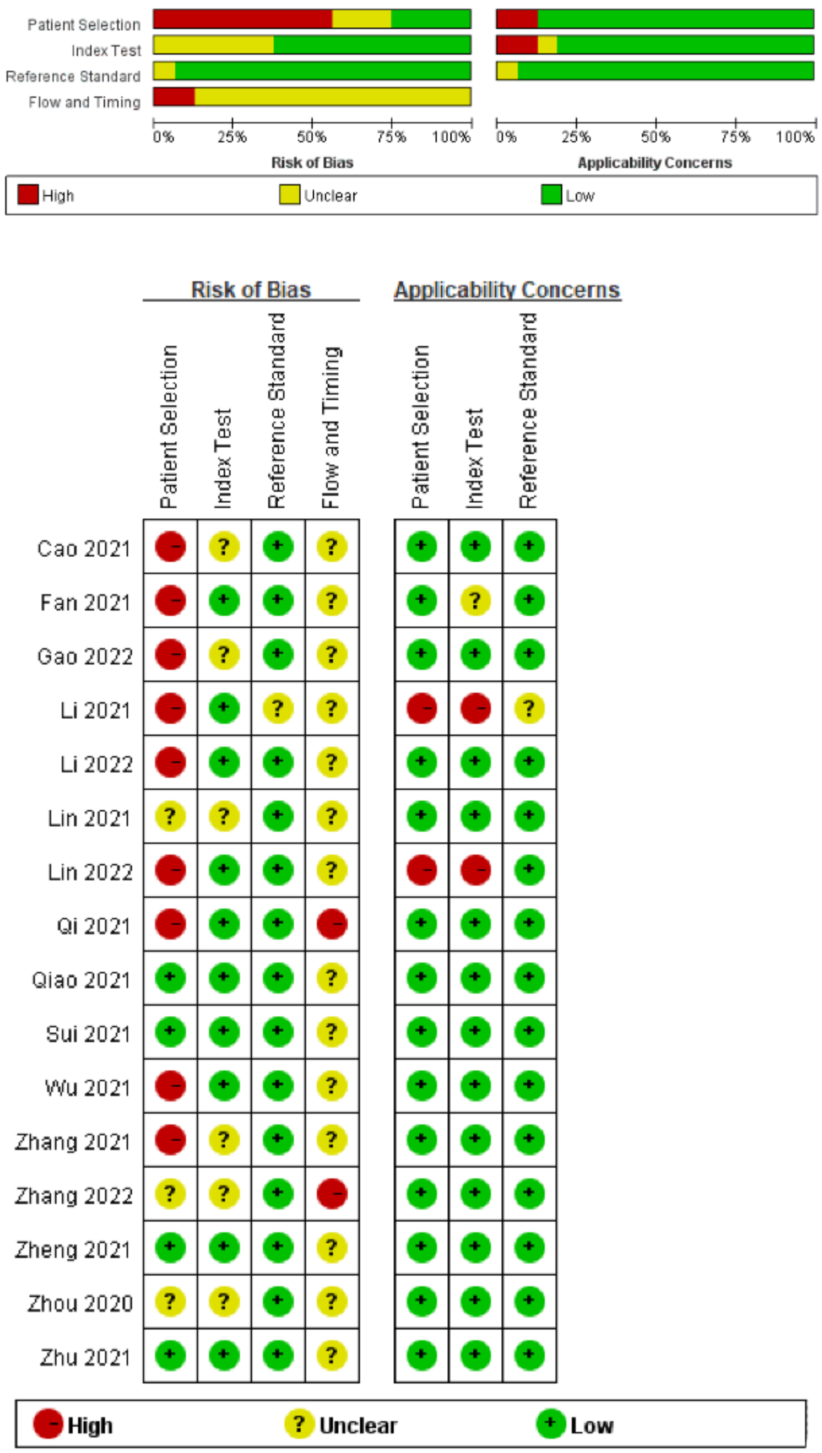

Supplementary Figure 1. Results of QUADAS-2 assessment for risk of bias in individual studies.
