# Supplementary material for: Diagnostic performance of C-TIRADS in malignancy risk stratification of thyroid nodules: A systematic review and meta-analysis
Source: Front Endocrinol (Lausanne). 2022 Sep 8;13:938961. doi: 10.3389/fendo.2022.938961 (PMC9492922; doi:10.3389/fendo.2022.938961)
Supplement: Supplementary file 2 [file Table_1.docx]

## Supplementary Tables

**Table S1.** The results of the sensitivity analysis.

| First author | Sensitivity (95% CI) | Specificity (95% CI) | DOR (95% CI) | LR+ (95% CI) | LR- (95% CI) | AUC (95% CI) |
| --- | --- | --- | --- | --- | --- | --- |
| Cao 2021 | 0.93(0.88-0.96) | 0.71(0.61-0.80) | 38.06(26.08-52.83) | 3.48(2.45-4.64) | 0.10(0.05-0.14) | 0.94(0.92-0.96) |
| Fan 2021 | 0.94(0.89-0.97) | 0.69(0.59-0.78) | 35.82(21.66-54.27) | 3.17(2.69-3.81) | 0.10(0.06-0.18) | 0.94(0.90-0.96) |
| Gao 2022 | 0.94(0.89-0.97) | 0.69(0.58-0.78) | 34.88(26.30-44.82) | 3.23(2.49-4.35) | 0.10(0.06-0.17) | 0.93(0.91-0.95) |
| Li 2021 | 0.94(0.90-0.97) | 0.69(0.59-0.78) | 35.02(26.62-42.46) | 3.13(2.06-4.19) | 0.09(0.06-0.13) | 0.94(0.92-0.95) |
| Li 2022 | 0.94(0.90-0.97) | 0.69(0.58-0.78) | 37.35(26.66-46.24) | 3.17(2.48-4.29) | 0.09(0.06-0.13) | 0.94(0.92-0.96) |
| Lin 2021 | 0.93(0.88-0.96) | 0.71(0.61-0.80) | 38.93(24.35-55.30) | 3.13(2.34-3.63) | 0.08(0.06-0.12) | 0.94(0.92-0.96) |
| Lin 2022 | 0.95(0.92-0.97) | 0.67(0.58-0.75) | 38.26(28.18-48.27) | 2.89(2.10-3.56) | 0.08(0.05-0.11) | 0.94(0.91-0.96) |
| Qi 2021 | 0.94(0.89-0.97) | 0.71(0.61-0.80) | 38.99(27.82-47.04) | 3.16(2.60-3.92) | 0.09(0.06-0.15) | 0.93(0.91-0.95) |
| Qiao 2021 | 0.93(0.88-0.96) | 0.72(0.62-0.80) | 35.28(23.19-43.52) | 3.54(2.95-4.14) | 0.12(0.07-0.20) | 0.92(0.88-0.95) |
| Sui 2021 | 0.94(0.89-0.97) | 0.68(0.58-0.77) | 37.91(27.43-59.47) | 2.94(2.19-3.83) | 0.08(0.04-0.13) | 0.94(0.92-0.97) |
| Wu 2021 | 0.94(0.89-0.97) | 0.71(0.60-0.80) | 40.71(28.75-52.02) | 3.31(2.68-4.33) | 0.08(0.06-0.12) | 0.94(0.92-0.96) |
| Zhang 2021 | 0.93(0.88-0.97) | 0.71(0.61-0.80) | 33.60(27.81-44.77) | 3.37(2.58-4.45) | 0.10(0.07-0.14) | 0.93(0.92-0.95) |
| Zhang 2022 | 0.93(0.88-0.96) | 0.70(0.60-0.79) | 35.41(24.55-53.27) | 3.26(2.52-4.11) | 0.10(0.06-0.16) | 0.93(0.91-0.96) |
| Zheng 2021 | 0.92(0.88-0.96) | 0.73(0.64-0.80) | 36.51(27.81-49.68) | 3.24(2.86-4.15) | 0.09(0.06-0.13) | 0.94(0.92-0.96) |
| Zhou 2020 | 0.94(0.89-0.97) | 0.70(0.59-0.79) | 33.86(19.28-48.80) | 3.24(2.42-4.03) | 0.10(0.06-0.19) | 0.93(0.89-0.95) |
| Zhu 2021 | 0.93(0.88-0.97) | 0.70(0.60-0.79) | 31.94(22.68-50.65) | 3.62(2.73-4.78) | 0.12(0.06-0.16) | 0.92(0.90-0.95) |

DOR, diagnostic odds ratio; LR+, positive likelihood ratio; LR-, negative likelihood ratio; AUC, area under curve; 95% CI, 95% confidence interval.
